# Supplementary figures and images for: Evaluation of first trimester maternal serum inhibin-A for preeclampsia screening
Source: PLoS One. 2023 Jul 10;18(7):e0288289. doi: 10.1371/journal.pone.0288289 (PMC10332599; doi:10.1371/journal.pone.0288289)

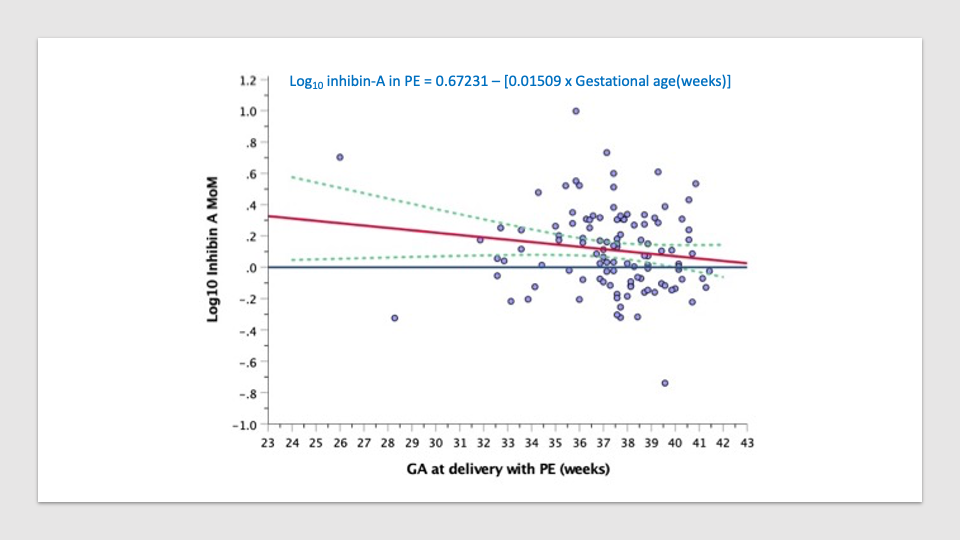

Supplement: S1 Fig — Dashed lines represent the observed expected mean level of log10 inhibin-A MoM and its 95% upper and lower confidence interval. (TIF) [file pone.0288289.s001.tif]
