# Supplementary material for: Evaluation of first trimester maternal serum inhibin-A for preeclampsia screening
Source: PLoS One. 2023 Jul 10;18(7):e0288289. doi: 10.1371/journal.pone.0288289 (PMC10332599; doi:10.1371/journal.pone.0288289)
Supplement: S1 Table — (DOCX) [file pone.0288289.s002.docx]

**S1 Table: Model derived from 1680 unaffected pregnancies used to estimate the expected ThermoFisher BRAHMS KRYPTOR log_10_ inhibin-A in at 11-13 weeks.**

| **Term** | **Estimate** |
| --- | --- |
| Intercept | 2.41729 |
| Gestational age in days -77 | –0.01204 |
| Maternal Age in years | +0.00522 |
| Maternal Weight in kg - 69 | –0.00535 |
| Parous without prior PE | –0.04223 |
