# Supplementary material for: Evaluation of first trimester maternal serum inhibin-A for preeclampsia screening
Source: PLoS One. 2023 Jul 10;18(7):e0288289. doi: 10.1371/journal.pone.0288289 (PMC10332599; doi:10.1371/journal.pone.0288289)
Supplement: S2 Table — (DOCX) [file pone.0288289.s003.docx]

**S2 Table: Observed log_10_ MoM biomarker distribution standard deviation and inter –biomarker correlations in women with and without preeclampsia.**

| **Log_10_ Biomarker** | **Pooled N=1792** | **Unaffected**  **N=1680** | **Pre-eclampsia N=112** |  |
| --- | --- | --- | --- | --- |
|  |  |  |  |  |
| **Distribution Standard Deviation** |  |  |  |  |
| MAP MoM | 0.0407 | 0.0395 | 0.0478 |  |
| UTPI MoM | 0.1106 | 0.1091 | 0.1295 |  |
| PlGF MoM | 0.1991 | 0.1940 | 0.2262 |  |
| Inhibin-A MoM | 0.2238 | 0.2108 | 0.2571 |  |
|  |  |  |  |  |
| **Correlations** |  |  |  |  |
| MAP MoM vs UTPI MoM | -0.0390 | -0.0402 | -0.1412 |  |
| MAP MoM vs PlGF MoM | -0.0976* | -0.0862* | 0.0863 |  |
| MAP MoM vs Inhibin-A MoM | 0.0679* | 0.0563* | 0.0243 |  |
| UTPI MoM vs PlGF MoM | -0.1961* | -0.1772* | -0.3376* |  |
| UTPI MoM vs Inhibin-A MoM | -0.0172 | -0.0165 | -0.0934 |  |
| PlGF MoM vs Inhibin-A MoM | -0.0360 | 0.0001 | -0.3498* |  |
|  |  |  |  |  |

**Footnote:** MoM: Multiple of median; MAP: Mean Arterial Pressure; UTPI: Uterine Artery Pulsatility Index; PlGF: Placental growth factor**,** *: statistical significance at p<0.05
