# Supplementary material for: Evaluation of first trimester maternal serum inhibin-A for preeclampsia screening
Source: PLoS One. 2023 Jul 10;18(7):e0288289. doi: 10.1371/journal.pone.0288289 (PMC10332599; doi:10.1371/journal.pone.0288289)
Supplement: Attachment — Submitted filename: Response to reviewers.docx [file pone.0288289.s004.docx]

**Response letter to reviewers’ and editors’ comment**

Dear editors and reviewers,

Thank you very much for your comments and suggestion. The manuscript was amended, and the following points were addressed.

**1)** What can be the reason for lower inhibin-A levels in parous women with no previous history of PE? This could be shortly discussed

**Response:** Thank you, the discussion based on this issue was addressed as following: “We have further demonstrated that there are lower inhibin-A levels in parous women without prior PE, which reduces the risk for developing PE in the current pregnancy. This finding may indicate that women with a previous unaffected pregnancy have the ability to accomplish adequate maternal adaptation to tolerate pregnancy-related stress in the previous pregnancy, thus providing a protective effect against the development of PE in the current pregnancy..”

**2)** Table 1: by looking at the differences between groups in many comparisons, several in the term PE vs control comparisons (e.g., IVF, nulliparous, etc.) could be significant. Please double check the numbers.

**Response:** Thank you, the description of baseline characteristics was expanded as following: “Compared with the unaffected pregnancies, women with PE had higher maternal age and BMI, higher rates of IVF conception, nulliparity and previous history of PE, and lower rate of history of previous pregnancy without PE. There was no difference in the rates of smoking, chronic hypertension, and SLE/APS between groups. Although there was a higher rate of pre-existing diabetes mellitus in the PE group, compared to the unaffected group, the total number of cases was small.”

**3)** It is stated that tests were considered statistically significant if the p-value was <0.05. What was the threshold after Bonferroni correction?

**Response:** Thank you, ANOVA with Bonferroni correction with threshold (p value<0.016) for multiple comparison was used. It was added to the manuscript.

**4)** It is stated that “inhibin-A in the currently used FMF triple test does not significantly improve the screening performance could be explained by the concept of diminishing marginal returns and the fact that inhibin-A levels correlate with PlGF levels in PE groups.” However, there was no testing of any correlation. Please perform the test or remove the statement.

**Response:** Thank you, S2 Table which demonstrated observed log_10_ MoM biomarker distribution standard deviation and inter-biomarker correlations in women with and without preeclampsia was added. It demonstrated significant correlation between inhibin-A and PlGF levels.

Best regards,

Sakita Moungmaithong
